# The Practical Application of the Röntgen Rays in Therapeutics and Diagnosis

**Published:** 1904-03

**Authors:** 


**The Practical Application of the Röntgen Rays in Therapeutics and Diagnosis.** By WILLIAM ALLEN PUSEY, M.D., AND EUGENE WILSON CALDWELL. Pp. 591. London: W. B. Saunders & Co. 1903.

The appearance of this bulky volume of nearly six hundred pages is a testimony to the growing importance of the X-rays in medical and surgical practice. The work is, as its title claims, eminently "practical" in character, and is a record mainly of the two authors' own personal experience, and is the more valuable in consequence.

The volume is divided into two parts—one of the authors writing on the technique of the X-rays and the other on their therapeutic use. The first is one of the best and most practical descriptions of the necessary apparatus and the mode of using it that we have met with, and can be cordially recommended to any worker on the subject. One of the best chapters is the one on fluoroscopy, in which the limitations of an examination by the fluorescent screen is rightly insisted on. It is so very easy "to overlook fractures where there is little or no displacement, and even foreign bodies if they are of no great size." Again, in the chapter on printing from the negative, a point is insisted on viz.: "It is impossible to get in a print all the detail which may be seen in an X-ray negative," a point not sufficiently taken into consideration by persons not accustomed to work radiography.

The second part of the volume, and the larger part, is given up to the therapeutics of the X-ray. The author states that his chief object is to present "facts" towards the elucidation of what the X-rays will do and what they will not. Accordingly after some excellent chapters on the pathological effects of the rays on the tissues of the body, he proceeds to take each disease, or set of diseases, in which the rays have been used therapeutically, and give the results that other workers have recorded, and then give detailed notes of cases (mostly illustrated with photographs) in which he has himself used the remedy; after which he sums up the results of his own and other workers' treatment in each case. The result of his work is to bear witness to the great value of the X-ray as a therapeutic agent. Thus in common with other observers he has been most successful in his treatment of rodent ulcer, here called cutaneous carcinoma; also in various forms of skin disease, etc. He also bears testimony to the wonderful anodyne effects of the rays in inoperable malignant disease, and not only this, but in some of the cases quoted there were some marvellous curative results; thus in five out of the eighteen cases which had been treated by the author there was "symptomatic cure."

Taking it altogether, this work is one of the best and most practical which has appeared on the subject, and can be thoroughly recommended to anyone interested in radiography.

